# *Hsa_circ_0060450* Negatively Regulates Type I Interferon-Induced Inflammation by Serving as miR-199a-5p Sponge in Type 1 Diabetes Mellitus

**DOI:** 10.3389/fimmu.2020.576903

**Published:** 2020-09-29

**Authors:** Lan Yang, Xiao Han, Caiyan Zhang, Chengjun Sun, Saihua Huang, Wenfeng Xiao, Yajing Gao, Qiuyan Liang, Feihong Luo, Wei Lu, Jinrong Fu, Yufeng Zhou

**Affiliations:** ^1^Institute of Pediatrics, Children's Hospital of Fudan University, Shanghai Key Laboratory of Medical Epigenetics, International Co-laboratory of Medical Epigenetics and Metabolism, Ministry of Science and Technology, Institutes of Biomedical Sciences, Fudan University, Shanghai, China; ^2^National Health Commission (NHC) Key Laboratory of Neonatal Diseases, Fudan University, Shanghai, China; ^3^Department of Pediatric Endocrinology and Inherited Metabolic Diseases, Children's Hospital of Fudan University, Shanghai, China

**Keywords:** type I interferon, SHP2, macrophage, type 1 diabetes mellitus, circular RNA

## Abstract

Circular RNAs (circRNAs) constitute a class of covalently circular non-coding RNA molecules formed by 5′ and 3′ end back-splicing. The rapid development of bioinformatics and large-scale sequencing has led to the identification of functional circRNAs. Despite an overall upward trend, studies focusing on the roles of circRNAs in immune diseases remain relatively scarce. In the present study, we obtained a differential circRNA expression profile based on microarray analysis of peripheral blood mononuclear cells (PBMCs) in children with type 1 diabetes mellitus (T1DM). We characterized one differentially expressed circRNA back-spliced from the MYB Proto-Oncogene Like 2 (*MYBL2*) gene in patients with T1DM, termed as *hsa_circ_0060450*. Subsequent assays revealed that *hsa_circ_0060450* can serve as the sponge of miR-199a-5p, release its target gene, Src homology 2 (SH2)-containing protein tyrosine phosphatase 2 (SHP2), encoded by the tyrosine-protein phosphatase non-receptor type 11 gene (*PTPN11*), and further suppress the JAK-STAT signaling pathway triggered by type I interferon (IFN-I) to inhibit macrophage-mediated inflammation, which indicates the important roles of circRNAs in T1DM and represents a promising therapeutic molecule in the treatment of T1DM.

## Introduction

Type 1 diabetes (T1DM) is an autoimmune disease, also known as insulin-dependent diabetes mellitus, owing to the complete absence of insulin secretion. During the pathogenesis of T1DM, insulin-producing pancreatic β cells are attacked by the immune system, resulting in destruction of islet function and decreased insulin secretion (1, 2). Immune tolerance mediated by Treg cells is outweighed by immune activation involving autoreactive CD8^+^ T cells, Th1 cells, and B cells (3). Furthermore, the expansion of IL-17-secreting Th17 cells also contributes to pancreatic inflammation in patients with T1DM (4, 5). In addition to adaptive immune cells, innate immune cells are involved in the onset of T1DM (6–8). Emerging evidence suggests that the absence of Toll-like receptor signaling in innate immune cells, caused by TIR-domain-containing adapter-inducing interferon-β (TRIF) deficiency, has a protective effect against diabetes in the non-obese diabetic (NOD) mouse via alteration of the gut microbiota and reduced immune cell activation (9).

Macrophages, known as inflammatory mediator-secreting immune cells, play a critical role in insulitis, and support autoimmune T cells to aggravate the infiltration of inflammatory cells during T1DM (10, 11). Macrophage migration inhibitory factor (Mif) knockout (KO) mice were found to express lower levels of costimulatory molecules in the streptozocin-induced diabetic mouse model and Toll-like receptors on the surface of their macrophages, leading to functional deficit of macrophages and impaired T cell activation, thereby alleviating pancreatic injury (12). Moreover, Unanue et al. have showed that islet-resident macrophages are in a persistent inflammatory state prior to signs of β cell pathology and their depletion significantly reduced the onset of diabetes (13, 14).

In addition to inflammation caused by bacterial infection, viral infection and subsequent IFN-I production are linked to T1DM pathogenesis (15–17). Viral infections can lead to apoptosis of pancreatic β cells and pancreas damage (18). Some viruses, such as coxsackievirus B1, parainfluenza, and enteroviruses initiated a devastating immune response in pancreatic β cells and other surrounding cells, resulting in the occurrence of fulminant T1DM through the induction of IFN-I and inflammation (19, 20). In addition, 2 independent longitudinal studies have showed the expression of IFN-I–induced genes in PBMCs isolated from children at risk for T1DM preceded onset of autoimmunity (21, 22). Moreover, some studies have confirmed that abrogation of IFN-I function significantly ameliorated the incidence of diabetes in NOD mice (23, 24). Marro et al. also found that macrophages promoted autoreactive CD8^+^ T cell infiltration into islets in the *Rip*-LCMV-GP T1DM model via IFN-I signaling, and abrogation of IFN-I signaling on macrophages significantly limited the onset of T1DM (25).

Circular RNAs (circRNAs) are a class of covalently circular non-coding RNA molecules (26, 27). Existing studies have shown circRNAs are generated mainly through three distinct mechanisms: exon skipping, RNA binding protein (RBP)-driven circularization, and intron pairing-driven circularization, which bring the 5′ and 3′ termini of mRNA into spatial proximity and induce back-splicing. The length of most human exonic circRNAs is <1,500 nucleotides (nt), and the median length is 500 nt (28). Compared with their homologous linear transcripts, circRNAs are not easily degraded by RNase R (29), and highly conserved between tissues, developmental stages, and cell types (30). By integrating large-scale deep-sequencing data sets, Zheng et al. annotated 14 867 human circRNAs in deepBase v2.0, 1260 of which are orthologous to mouse circRNAs (31). Furthermore, some circRNAs have been shown to exert important physiological functions. In 2013, Memczak et al. found that there are 63 miR-7 binding sites within the circular transcript *ciRS-7* sequence of the *CDR1as* gene (30). Hansen et al. also found that circRNA Sry can act as a “sponge” to adsorb miR-138 (32). Substantial evidence has been published describing the competitive endogenous RNA (ceRNA) mechanism, which is the most widely studied mechanism in circRNA research. Through adsorption of microRNAs, circRNA reduces the quantity of free microRNAs in the cytoplasm and releases target genes of microRNAs, leading to occurrence or inhibition of certain diseases.

Notably, despite an overall upward trend, circRNA studies focusing on immune diseases are relatively scarce. In the present study, we obtained a differential circRNA expression profile based on microarray analysis of PBMCs in children with T1DM. We characterized an upregulated circRNA, back-spliced from MYB Proto-Oncogene Like 2 (*MYBL2*) in patients with T1DM, termed as *hsa_circ_0060450*. Subsequent assays revealed that *hsa_circ_0060450* can serve as the sponge of miR-199a-5p, releasing its target Src homology 2 (SH2)-containing protein tyrosine phosphatase 2 (SHP2) encoded by tyrosine-protein phosphatase non-receptor type 11 gene (*PTPN11*) in macrophages, and further suppress the JAK-STAT signaling pathway triggered by IFN-I to inhibit macrophage-mediated inflammation.

## Materials and Methods

### Human Normal and T1DM Samples

Peripheral blood samples of T1DM patients and healthy controls were collected from the Children's Hospital of Fudan University, Shanghai, China. The follow-up PBMCs were separated by Ficoll-Hypaque (GE Healthcare) within 24 h after collection. Human samples were obtained with informed consents from their parents, and the study was approved by the Research Ethics Board of the Children's Hospital of Fudan University.

### Microarray Analysis

PBMCs of T1DM patients and healthy controls were separated and performed SBC Human (4^*^180K) ceRNA microarray analysis (Shanghai Biotechnology Corporation, China). In brief, total RNA was extracted and purified using miRNeasy Mini Kit (QIAGEN, GmBH, Germany), followed by amplification and label using Low Input Quick Amp Labeling Kit, One-Color (Agilent technologies, Santa Clara, CA, USA). Each slide was hybridized with Cy3-labeled cRNA using Gene Expression Hybridization Kit (Agilent technologies, Santa Clara, CA, USA) in Hybridization Oven. After hybridization, slides were washed in staining dishes with Gene Expression Wash Buffer Kit (Agilent technologies, Santa Clara, CA, USA), and were then scanned by Agilent Microarray Scanner (Agilent technologies, Santa Clara, CA, USA). Finally, data were extracted with Feature Extraction software 10.7 (Agilent technologies, Santa Clara, CA, USA), and raw data were normalized by Quantile algorithm. Fold change > 2.0 and *P* < 0.05 was the criterion to screen differentially expressed circRNAs between T1DM patients and healthy controls. Microarray data have been deposited in the Gene Expression Omnibus (GEO) under accession numbers GSE133225 (submitted in other draft).

### Cell Culture and Treatment

Human THP1 and HEK-293T cell lines were obtained from the American Type Culture Collection (ATCC, Manassas, VA, USA). THP1 cells were maintained in RPMI-1640 medium with inactivated 10% fetal bovine serum (FBS, Gibco, Gaithersburg, MD, USA) and 1% penicillin-streptomycin (Gibco, Gaithersburg, MD, USA). HEK-293T cells were cultured in Dulbecco's modified Eagle's medium (DMEM, Gibco, Gaithersburg, MD, USA) supplemented with 10% FBS and 1% penicillin-streptomycin. Cells were grown at 37°C in a 5% CO_2_ humidified incubator. THP1-derived macrophages were induced using THP1 cells treated with 50 ng/ml phorbol 12-myristate 13-acetate (PMA) for 48 h. Chemically synthesized miR-199a-5p mimics, miR-133a-3p mimics, miR-133b mimics, *hsa_circ_0060450* siRNA-1 and *hsa_circ_0060450* siRNA-2 (200 nM, GenePharma, Shanghai, China) were transiently transfected into macrophages using Lipofectamine RNAiMAX reagent (Invitrogen, USA), according to the standard protocol. After 48 h of transfection, macrophages were treated with type I interferon (1,000 units/ml, PBL Assay Science, USA). The sequences of siRNAs, microRNA mimics and NC are listed in Supplementary Table 1.

### RNA Extraction and Reverse Transcription Quantitative PCR (RT-qPCR)

Total RNA was extracted using TRIzol (Invitrogen, USA) followed by reverse transcription using the PrimeScript II 1st Strand cDNA Synthesis Kit (Takara, Japan), according to the manufacturer's instructions. Real-time quantitative PCR reaction was performed with SYBR® Premix Ex Taq™ II (Takara, Japan) using the Roche 480 Real Time PCR System. We used β-actin as internal control and calculated fold change via the 2^−ΔΔ*CT*^ method. The primer pairs used for qPCR are listed in Supplementary Table 2.

### Nuclear and Cytoplasmic Separation

Nuclear and cytoplasmic separation assay was conducted using the Nuclear and Cytoplasmic Extraction Kit (CWBiotech, Beijing, China) according to the manufacturer's protocol. Briefly, 1 × 10^7^ cells were centrifuged at 3 × 10^3^ rpm for 5 min, resuspended in 500 μl of ribonuclease inhibitor added-Nc-buffer A, and incubated on ice for 20 min. Then, 27.5 μl of Nc-Buffer B was added, and incubated on ice for 1 min. The mixtures were then centrifuged at 12,000 g, 4°C for 15 min. The supernatants (cytoplasmic component) were mixed with TRIzol reagent for RNA extraction. Next, the nuclear pellets were resuspended with 250 μl of ribonuclease inhibitor added-Nc-buffer C, and then mixed with TRIzol reagent for RNA extraction. RNA extraction and RT-qPCR were performed as previously described.

### Western Blot

Cell lysates were prepared with RIPA buffer (Thermo Fisher, USA); then, total protein was quantified with the Protein BCA Assay Kit (Bio-Rad, USA), and denatured at 100°C for 10 min. Next, 10% sodium dodecyl sulfate polyacrylamide gel electrophoresis (SDS-PAGE) was used to separate the protein (20 μg). The proteins were transferred to polyvinylidene fluoride (PVDF) membranes (Millipore Corporation, USA) and blocked in 5% bovine serum albumin (BSA) at room temperature (RT) for 1 h, followed by incubation overnight at 4°C with the rabbit anti-β-tubulin antibody (1:5000, Abcam, USA), anti-p-Stat1 antibody (Y701, 1:1000, Cell Signaling Technology, USA), anti-p-Stat3 antibody (Y705, 1:1000, Cell Signaling Technology, USA), anti-Stat1 antibody (1:1000, Cell Signaling Technology, USA), anti-Stat3 antibody (1:1000, Cell Signaling Technology, USA) and anti-SHP2 antibody (1:500, Santa Cruz, USA). Subsequently, after washing with tris buffered saline solutions with 0.1% Tween-20 (TBST) for three times, the membranes were incubated with horseradish peroxidase (HRP)-conjugated goat-anti-rabbit antibody (1:5000, Cell Signaling Technology, USA) at RT for 1 h. Finally, after washing again with TBST solution for three times, the bands were immersed in chemiluminescent HRP substrate (Millipore Corporation) determination and imaged with a Molecular Imager® (Bio-RAD, ChemiDocTM XRS+ Imaging System).

### Dual-Luciferase Reporter Assay

The wild type (WT) and mutant *hsa_circ_0060450* fragments and SHP2 3′-UTR containing corresponding miRNAs binding sites were fused into Renilla luciferase gene (hRluc) included psicheck2 vector (Promega, Madison, WI, USA) using the *Xho* I and *Not* I restriction sites. The fused recombinant vectors were verified by sanger sequencing. The PCR primers for WT and mutant *hsa_circ_0060450* fragments and SHP2 fragments are listed in Supplementary Table 2.

HEK-293T cells were seeded into a 96-well plate at a density of 10,000 cells/well. The recombinant vector or empty vector and miR-199a-5p, miR-133a-3p, miR-133b mimics, or NC were co-transfected simultaneously into HEK-293T cells using Lipofectamine 2000 (Invitrogen, CA, USA). Luciferase activity was determined 24 h after transfection using a dual luciferase reporter assay kit (Promega, USA).

### RNase R Treatment

A total of 5 μg of RNA extracted from cells using TRIzol reagent was incubated at 37°C for 30 min with RNase R (20 U/μl, Lucigen, USA) treatment according to the manufacturer's instructions; the control group was treated under RNase R-free conditions. The remaining RNA was then purified with phenol/chloroform mixture (5:1, Sigma-Aldrich, USA). Subsequent RNA extraction and RT-qPCR was performed as previously described.

### Actinomycin D Treatment

THP1 cells were seeded into a 24-well plate at a density of 120,000 cells/well with PMA (50 ng/ml) stimulation for 48 h. Then, total RNA was harvested at 0, 3, 6, 12, and 24 h, with or without actinomycin D (ActD) treatment (1 μg/ml). Subsequent RNA extraction and RT-qPCR was performed as previously described.

### Statistical Analysis

Results from three independent, replicate experiments were obtained to confirm the reproducibility of each experiment. Bars represent the means ± SEM. The two-tailed Student's *t*-tests was used for comparisons between two groups, and one-way analysis of variance (ANOVA) was used for comparisons of multiple groups. A *p*-value of <0.05 was considered to indicate statistically significant data. Statistical analyses were performed with SPSS v.19.0 software and visualized data were obtained with Graphpad prism 7.0 software.

## Results

### *Hsa_circ_0060450* Is Upregulated in PBMCs of T1DM Patients

**Figure 1:**
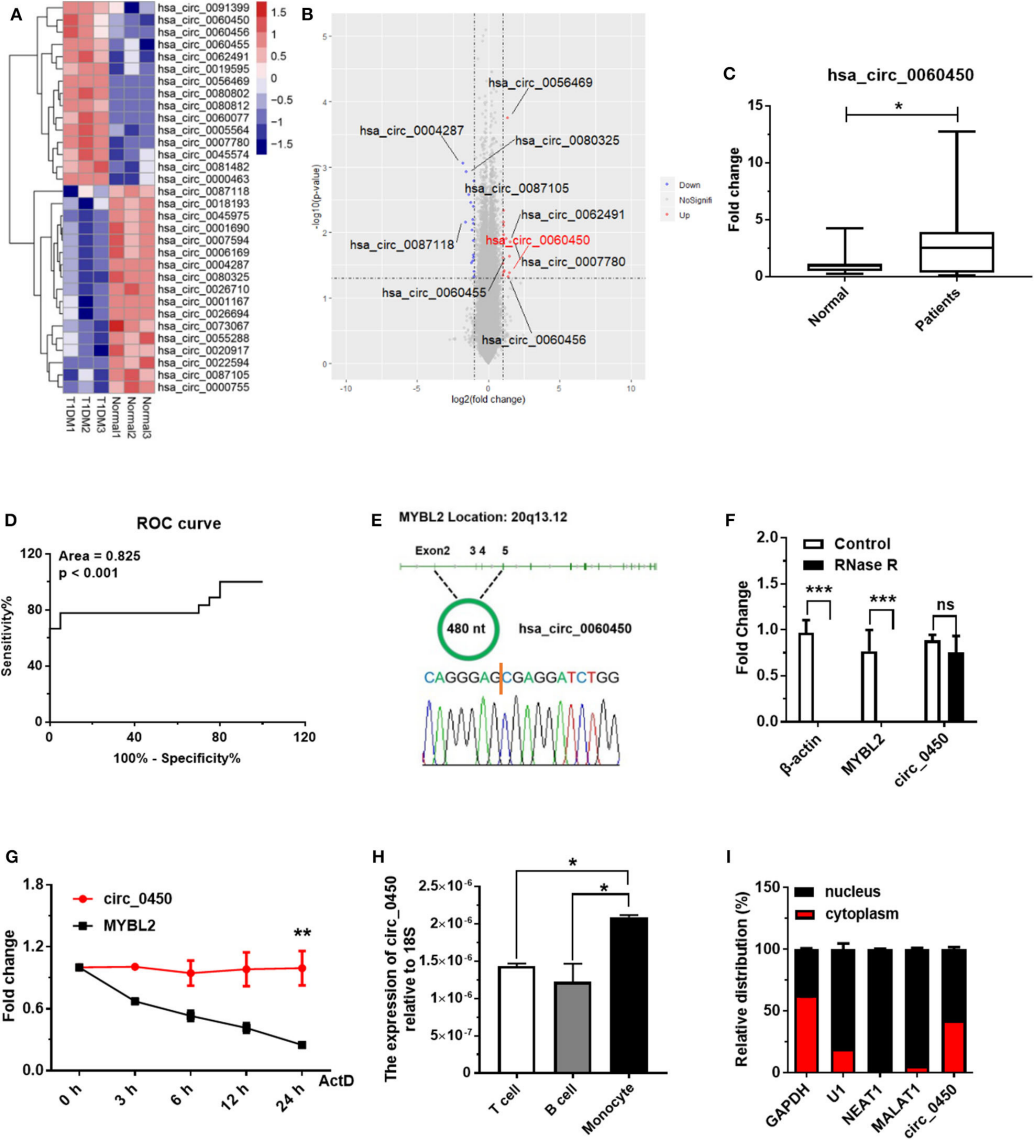
*Hsa_circ_0060450* is upregulated in the PBMCs of T1DM patients. **(A)** Heatmap of circRNAs with significantly altered expression in the PBMCs of 3 normal controls and 3 T1DM patients. **(B)** Volcano plot of circRNAs with significantly altered expression in the PBMCs of 3 normal controls and 3 T1DM patients. **(C)** The expression of *hsa_circ_0060450* in PBMCs of 20 children with T1DM and 20 healthy controls, detected by RT-qPCR. **(D)** Receiver operating curve (ROC) analysis of *hsa_circ_0060450* levels in the study population. **(E)** The genomic loci of the *MYBL2* and *hsa_circ_0060450*. The expression of *hsa_circ_0060450* was detected by RT-PCR and validated by Sanger sequencing. **(F)** RT-qPCR analyses of β-actin, *MYBL2*, and *hsa_circ_0060450* after treatment with RNase R in THP1-derived macrophages. **(G)** RT-qPCR analyses of *MYBL2* and *hsa_circ_0060450* after treatment with actinomycin D at the indicated time points in THP1-derived macrophages. **(H)** The expressions of *hsa_circ_0060450* in T cells, B cells and monocytes of human PBMCs, detected by RT-qPCR. **(I)** The relative distribution of *hsa_circ_0060450* in the nucleus and cytoplasm in THP1-derived macrophages. *circ_0450* is the abbreviation of *hsa_circ_0060450*. ActD, actinomycin D. Data represent means ± SEM. *p < 0.05, **p < 0.01, ***p < 0.001. ns, not significance.

In a pilot study, we obtained the differential circRNA expression profile based on microarray analysis of PBMCs in children with T1DM and age-matched healthy controls. We identified 15 upregulated and 17 downregulated circRNAs between T1DM patients and healthy controls (*n* = 3, fold change > 2, and *p* < 0.05) (Figure 1A). Next, we verified the expressions of the top 10 circRNAs (Figure 1B) with the highest differential expression fold in a larger population of clinical samples of PBMCs collected from 20 T1DM children and 20 healthy controls by RT-qPCR, and the results showed *hsa_circ_0060450* was detectable and significantly upregulated in PBMCs of T1DM patients than controls (Figure 1C). In addition, through ROC curve analysis, we found that the expression of *hsa_circ_0060450* in PBMCs could discriminate normal subjects and T1DM patients (Figure 1D). Using RT-PCR with divergent primers and DNA sequencing, we confirmed the back-splicing site sequences of *hsa_circ_0060450* (Figure 1E). The full-length sequences of *hsa_circ_0060450* were listed in Supplementary Figure 1. After treatment with RNase R, hsa_circ_0060450 did not degrade readily compared with its cognate linear transcript *MYBL2* (Figure 1F). Actinomycin D assay also revealed that *hsa_circ_0060450* is more stable than its linear transcript *MYBL2*, with a half-life exceeding 24 h (Figure 1G). Furthermore, to investigate the roles of *hsa_circ_0060450* in T1DM pathogenesis, we separated human PBMCs to detect the expression of *hsa_circ_0060450* in T cells, B cells and monocytes, respectively, and found the expression of *hsa_circ_0060450* is higher in monocytes compared with T cells and B cells (Figure 1H). Given that monocytes can transform into macrophages to participate in pancreas inflammation, we next mainly explored the function of *hsa_circ_0060450* in macrophages. Subsequently, we performed nuclear and cytoplasmic separation experiments in THP1-derived macrophages, and found that ~40% of *hsa_circ_0060450* is located in the cytoplasm, with the remaining proportion in the nucleus (Figure 1I).

### *Hsa_circ_0060450* Negatively Regulates IFN-I-Induced Inflammation Through the JAK-STAT1/3 Pathway

**Figure 2:**
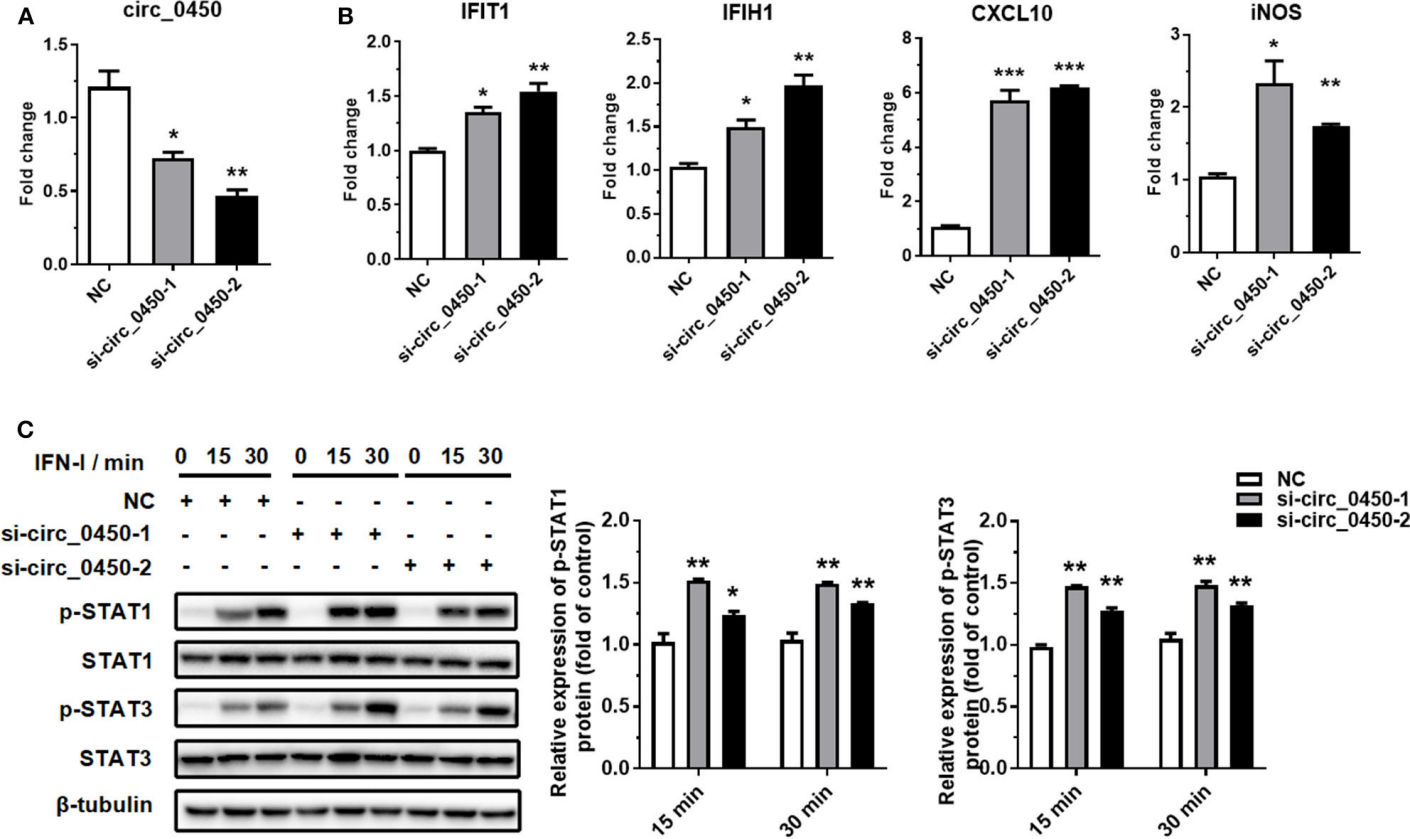
Silencing of *hsa_circ_0060450* promotes IFN-I-induced inflammation through the JAK-STAT1/3 pathway. **(A)** RT-qPCR analyses of *hsa_circ_0060450* to confirm the silencing efficiencies of two siRNAs. **(B)** RT-qPCR analyses of *IFIT1, IFIH1, CXCL10*, and *iNOS* under IFN-I stimulation after treatment with two *hsa_circ_0060450* siRNAs. **(C)** STAT1 and STAT3 protein phosphorylation assessment at 15 and 30 min of IFN-I stimulation after treatment with two *hsa_circ_0060450* siRNAs. NC, negative control. Data represent means ± SEM. *p < 0.05, **p < 0.01, ***p < 0.001.

To further explore whether *hsa_circ_0060450* plays a role in IFN-I-induced inflammation, we designed two small interfering RNAs (siRNAs) to silence *hsa_circ_0060450* expression in THP1-derived macrophages (Figure 2A). Notably, the two siRNAs were targeted specifically to the back-splicing site of *hsa_circ_0060450*, ensuring that only *hsa_circ_0060450* was knocked down and its cognate linear transcript *MYBL2* was not targeted. RT-qPCR assay showed that *hsa_circ_0060450* silencing resulted in significant upregulation of the expressions of *IFIT1, IFIH1, CXCL10*, and *iNOS* under IFN-I stimulation (Figure 2B). We then performed Western blotting assay to investigate the effect of *hsa_circ_0060450* on the JAK-STAT1/3 pathway activated by IFN-I. Experimental results showed that *hsa_circ_0060450* silencing promoted STAT1 and STAT3 protein phosphorylation after 15 min and 30 min of IFN-I stimulation (Figure 2C). This was indicative of the inhibitory action of *hsa_circ_0060450* on IFN-I-induced inflammation. Collectively, these results suggest that *hsa_circ_0060450* negatively regulates IFN-I-induced inflammation through the JAK-STAT1/3 pathway.

### *Hsa_circ_0060450* Serves as a Sponge of miR-199a-5p to Inhibit Inflammation Induced by IFN-I

**Figure 3:**
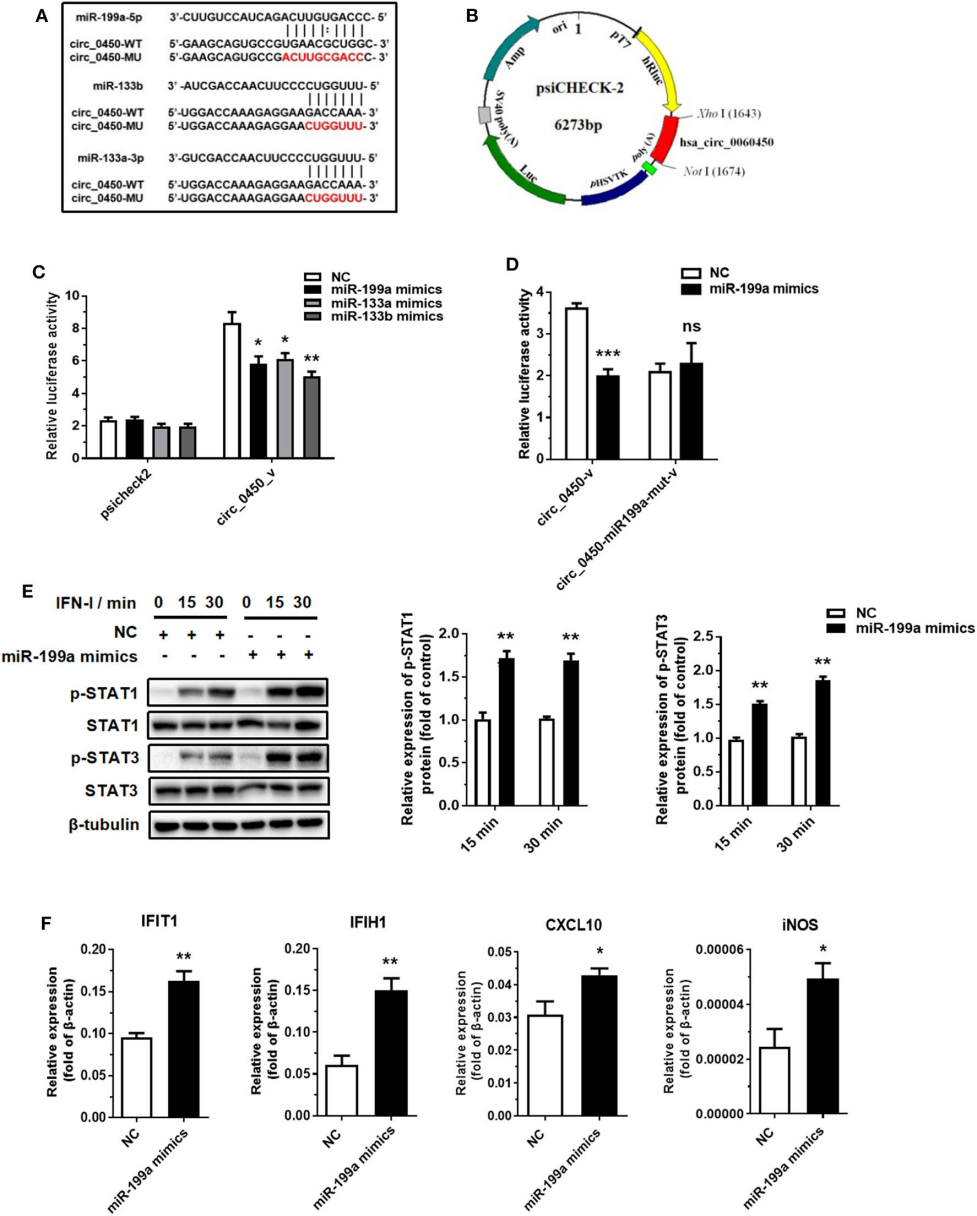
*Hsa_circ_0060450* serves as a sponge of miR-199a-5p. **(A)** The predicted target sequence of miR-199a-5p, miR-133a or miR-133b in *hsa_circ_0060450* and corresponding mutant target sequence for binding sites-mutant luciferase assay. **(B)** The construction of *hsa_circ_0060450* fragment-containing psicheck2 luciferase vector. **(C)** A luciferase reporter assay was used to detect the luciferase activity of 293T cells co-transfected with blank psicheck2 vector or psicheck2 recombinant vector containing *hsa_circ_0060450* fragment and miR-199a-5p, miR-133a, miR-133b mimics, or NC. **(D)** A luciferase reporter assay was used to detect the luciferase activity of 293T cells co-transfected with psicheck2 recombinant vector containing mutant *hsa_circ_0060450* fragment and miR-199a-5p or NC. **(E)** STAT1 and STAT3 protein phosphorylation assessment and quantification analyses at 15 and 30 min of IFN-I stimulation after treatment with miR-199a-5p mimics. **(F)** RT-qPCR analyses of *IFIT1, IFIH1, CXCL10*, and *iNOS* under IFN-I stimulation after treatment with miR-199a-5p mimics. NC, negative control. circ_0450-v, psicheck2 recombinant vector containing WT *hsa_circ_0060450* fragment. circ_0450-miR199a-mut-v, psicheck2 recombinant vector containing miR-199a-5p binding site-mutant *hsa_circ_0060450* fragment. Data represent means ± SEM. *p < 0.05, **p < 0.01, ***p < 0.001. ns, not significance.

Recent evidence suggests that exonic circRNAs within the cytoplasm can serve as a “sponge” of microRNAs. Therefore, we determined to investigate whether *hsa_circ_0060450* adsorbs microRNAs to exert its function. We first selected several microRNAs that have been studied for their pro-inflammatory effects, and among these microRNAs, miR-199a-5p, miR-133a-3p and miR-133b were predicted to have binding sites to *hsa_circ_0060450* by RNA22 database (https://cm.jefferson.edu/rna22/Interactive/) (Figure 3A). Next, we performed luciferase assay by constructing *hsa_circ_0060450* fragment-containing psicheck2 recombinant vectors (Figure 3B). Results showed that the luciferase activity of the group co-transfected with miR-199a-5p mimics and psicheck2 recombinant vector containing *hsa_circ_0060450* fragment, as well as that of the other two groups co-transfected with miR-133a or miR-133b mimics and psicheck2 recombinant vector containing *hsa_circ_0060450* fragment, was significantly reduced (Figure 3C). We also performed luciferase assay to confirm the target sequence of miR-199a-5p, miR-133a or miR-133b in *hsa_circ_0060450* by constructing mutant *hsa_circ_0060450* fragment-containing psicheck2 recombinant vectors (Figure 3A). Results showed that the luciferase activity of the group co-transfected with miR-199a-5p, miR-133a or miR-133b mimics and corresponding *hsa_circ_0060450*-mutant vector was not changed compared with the NC group (Figure 3D and Supplementary Figure 2). Furthermore, we investigated the pro-inflammatory effects of these three microRNAs in THP1-derived macrophages. The Western blotting results suggested that miR-199a-5p overexpression promoted STAT1 and STAT3 protein phosphorylation (Figure 3E); however, the overexpression of miR-133a and miR-133b inhibited STAT1 and STAT3 protein phosphorylation after IFN-I stimulation (Supplementary Figures 3A,B). Of note, RT-qPCR assay indicated that miR-199a-5p overexpression significantly upregulated the expressions of *IFIT1, IFIH1, CXCL10*, and *iNOS* under the stimulation of IFN-I (Figure 3F), consistent with the pro-inflammatory features of miR-199a as reported previously (33, 34), and miR-133a or miR-133b overexpression significantly reduced the expressions of *IFIT1, IFIH1, CXCL10*, and *CCL5* under the stimulation of IFN-I (Supplementary Figure 3C). To summarize, *hsa_circ_0060450* may serve as a sponge of miR-199a-5p to inhibit inflammation induced by IFN-I.

### miR-199a-5p Promotes IFN-I-Induced Inflammation by Targeting SHP2

**Figure 4:**
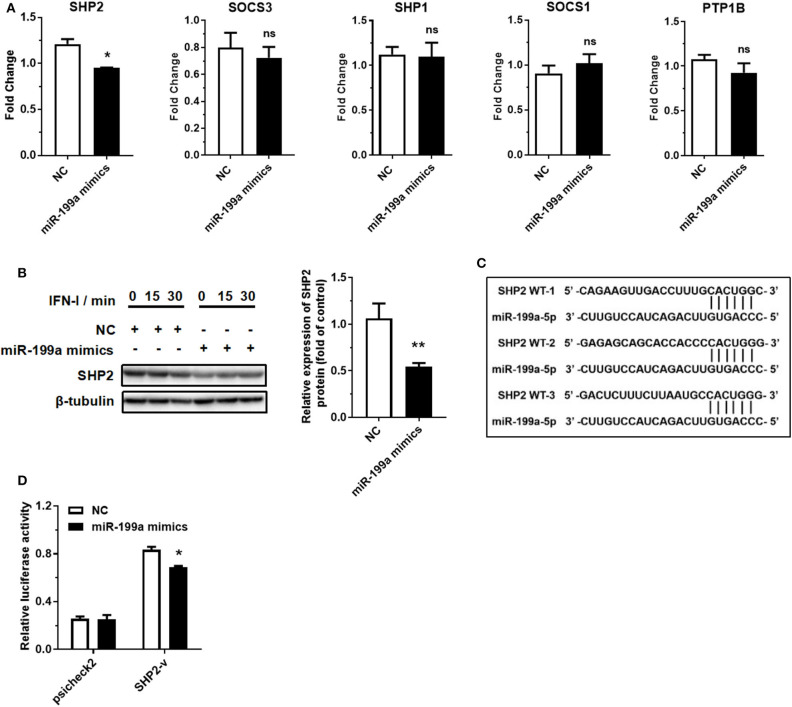
miR-199a-5p promotes IFN-I-induced inflammation by targeting SHP2. **(A)** RT-qPCR analyses of five predicted miR-199a-5p target genes after treatment with miR-199a-5p mimics. **(B)** SHP2 protein assessment at 0, 15, and 30 min of IFN-I stimulation after treatment with miR-199a-5p mimics. **(C)** Three miR-199a-5p binding sites within SHP2 3′ UTR predicted using the starBase database. **(D)** Luciferase activities of 293T cells co-transfected with blank psicheck2 vector or psicheck2 recombinant vector containing SHP2 3′ UTR fragment and miR-199a-5p mimics or NC. NC, negative control. SHP2-v, psicheck2 recombinant vector containing SHP2 3′ UTR fragment. Data represent means ± SEM. *p < 0.05, **p < 0.01. ns, not significance.

MicroRNAs can bind to the 3′ UTR of their targets to suppress translation or accelerate mRNA degradation (35, 36). miR-199a-5p may act as a pro-inflammatory factor by binding to its target to promote JAK-STAT1/3 pathway activation. We then selected five typical negative regulators of JAK-STAT pathway through a survey of the literature: SHP2, SOCS3, SHP1, SOCS1, and PTP1B (37). RT-qPCR assay demonstrated that the overexpression of miR-199a-5p reduced the expression of SHP2 at mRNA level, but not that of the other four negative regulators (Figure 4A). Western blotting assay also showed that overexpression of miR-199a-5p reduced the expression of SHP2 at protein level (Figure 4B). SHP2 was predicted to contain three miR-199a-5p binding sites according to the starBase database (http://starbase.sysu.edu.cn/index.php) (Figure 4C), and subsequent luciferase assay showed that the luciferase activity of the group co-transfected with miR-199a-5p mimics and part SHP2 sequence-containing psicheck2 vector was significantly reduced (Figure 4D). These findings suggest that SHP2 is a target of miR-199a-5p.

### *Hsa_circ_0060450* Upregulates SHP2 Expression by Adsorbing miR-199a-5p

**Figure 5:**
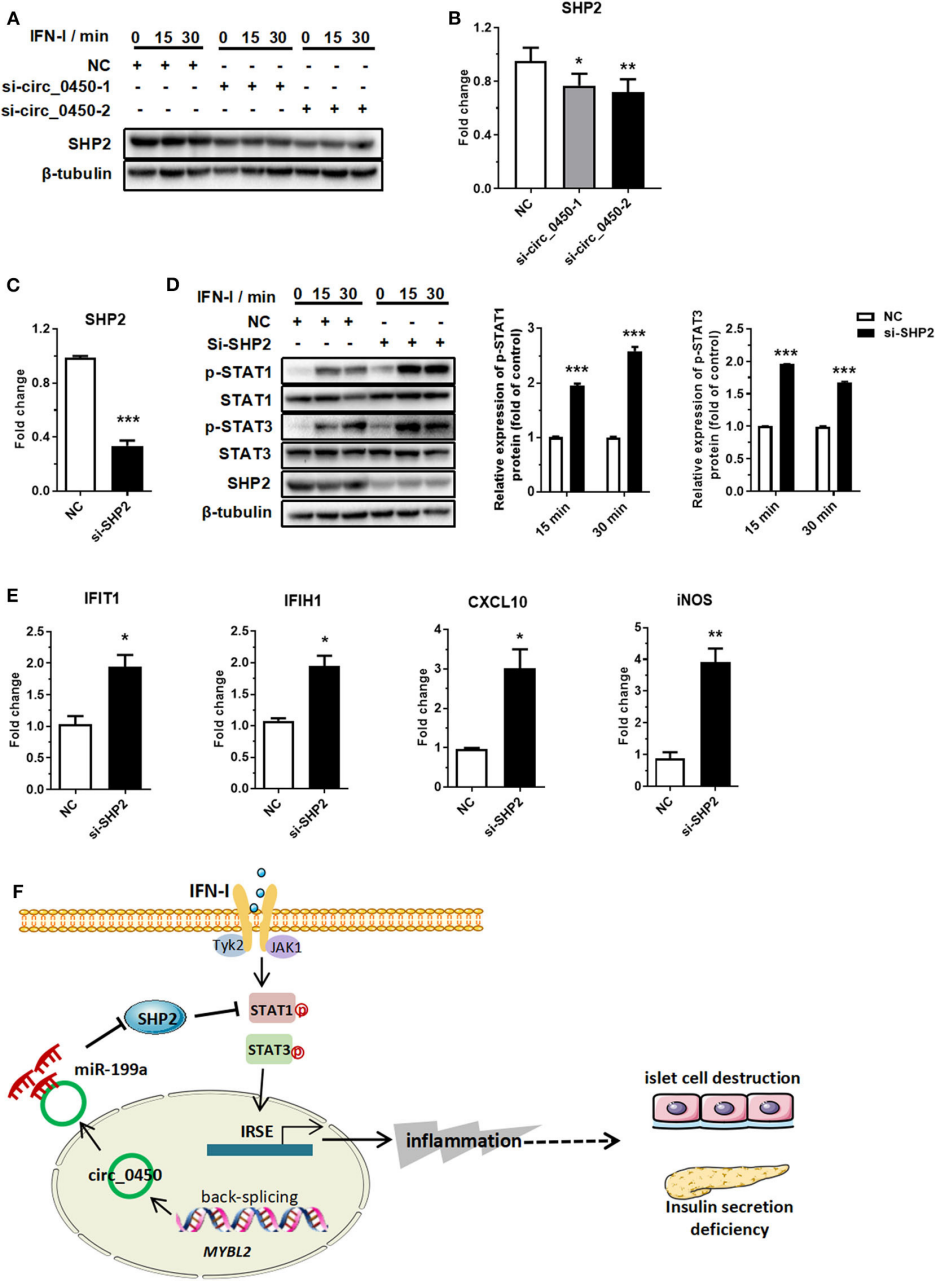
*Hsa_circ_0060450* upregulates SHP2 by adsorbing miR-199a-5p. **(A)** SHP2 protein assessment at 0, 15, and 30 min of IFN-I stimulation after treatment with two *hsa_circ_0060450* siRNAs. **(B)** RT-qPCR analyses of SHP2 expression after treatment with two *hsa_circ_0060450* siRNAs. **(C)** RT-qPCR analysis of SHP2 expression to confirm the silencing efficiency of the SHP2 siRNA. **(D)** STAT1 and STAT3 phosphorylation level assessment at 15 and 30 min of IFN-I stimulation after treatment with the SHP2 siRNA. **(E)** RT-qPCR analyses of *IFIT1, IFIH1, CXCL10*, and *iNOS* under IFN-I stimulation after treatment with the SHP2 siRNA. **(F)** A schematic model of the function of *hsa_circ_0060450* in IFN-I-stimulated macrophages and in T1DM. NC, negative control. Data represent means ± SEM. *p < 0.05, **p < 0.01, ***p < 0.001.

Further, we performed an in-depth investigation of the *hsa_circ_0060450*-miR-199a-5p-SHP2 axis. RT-qPCR and Western blotting results showed that *hsa_circ_0060450* silencing depressed SHP2 expression at both mRNA and protein levels (Figures 5A,B). Subsequently, we designed two SHP2-siRNAs to silent the expression of SHP2 (Figure 5C), and further to verify the inhibitory effect of SHP2, as a typical dephosphorylation enzyme, on IFN-I-induced JAK-STAT1/3 pathway activation. Western blotting results showed SHP2 silencing promoted STAT1 and STAT3 protein phosphorylation after IFN-I stimulation (Figure 5D). In addition, silencing SHP2 boosted the expressions of IFN-I-induced genes, including *IFIT1, IFIH1, CXCL10*, and *iNOS* (Figure 5E).

## Discussion

In this study, we obtained a differential circRNA expression profile based on microarray analysis of PBMCs in children with T1DM. We then characterized one circRNA, back-spliced from *MYBL2*, termed as *hsa_circ_0060450*, which exhibited upregulated expression in patients with T1DM. Subsequent assays revealed that *hsa_circ_0060450* serves as a “sponge” of miR-199a-5p to inhibit IFN-I-induced inflammation (Figure 5F). miR-199a-5p was reported to exert various biological functions, and existing studies have indicated the potential roles of miR-199a-5p in the pathogenesis of diabetes. Lin et al. demonstrated that miR-199a-5p is upregulated in pancreatic β cells in response to high glucose and promotes apoptosis and ROS generation in type 2 diabetes (38). In addition, a bioinformatics analysis showed that mmu-miR-199a-5p is closely related to inflammatory response, and insulin signal pathway in the liver of diabetic mice (39). SHP2 is a widely expressed SH2 domain-containing protein tyrosine phosphatase (40). Although sufficient evidence has shown that SHP2 is involved in tumorigenesis, its roles in immune regulation have also been gradually elucidated. Shuai et al. found SHP2 negatively regulated the IFN-induced JAK1/STAT1 pathway by dephosphorylating STAT1 (37). Wu et al. also demonstrated that phosphorylation at the tyrosine residue Tyr701 of STAT1 induced by IFN-α was enhanced and prolonged in SHP2-deletion cells (41). In addition, Zhang et al. and Ke et al. observed similar negative regulation on STAT3 in SHP2 knockout mice (42, 43). More recently, Krajewska et al. showed that mice with a neuron-specific, conditional SHP2 deletion developed obesity and diabetes-associated complications, including hyperglycemia, insulin resistance, etc. This suggests a protective role of SHP2 in diabetes (44). Despite these existing studies, the present study is the first, to our knowledge, to investigate the role of *hsa_circ_0060450*-miR-199a-5p-SHP2 axis in IFN-I-induced inflammation in T1DM.

Unexpectedly, even if showing an upward trend in the PBMCs of T1DM patients, *hsa_circ_0060450* did not prevent the occurrence of pancreatic inflammation and T1DM. Possibly, we consider that *hsa_circ_0060450* is produced reactively during pancreatic inflammation, but the amount produced in the body is not sufficient to inhibit macrophage inflammation. In addition, there may be other circRNAs or other types of molecules in T1DM patients that antagonistically inhibit anti-inflammatory effect of *hsa_circ_0060450*. Therefore, it is necessary to administer a larger dose of exogenous *hsa_circ_0060450* to achieve a certain effect of alleviating inflammation and treating T1DM.

Generally, circRNAs are classified into three types: exon circular RNA (ecircRNA), circular intronic RNA (ciRNA), and exon-intron circRNA (EIciRNA) (45). EcircRNA mainly exists in the cytoplasm, serving as a “sponge” of microRNAs, while the other two circRNAs are confined to the nucleus owing to the presence of introns (46–48). Zhang et al. demonstrated that intron-derived circular RNA ci-ankrd52 exists in the nucleus and cis-regulates transcription of its parental gene *ANKRD52* via the catalytic effect of RNA Pol II (47). Li et al. subsequently found that intron-exon circRNA circEIF3J and circPAIP2 form an RNA-protein complex with U1SNP and RNA Pol II through RNA-RNA interaction to cis-regulate the transcription of their parental genes (46). The parental gene of *hsa_circ_0060450, MYBL2*, is a transcription factor of the MYB family, and was first studied to be a physiological regulator of cell cycle progression, cell differentiation and cell survival, but recently it was frequently found deregulation in numerous cancer entities and significantly drove cancer initiation and progression (49). Moreover, a new non-synonymous SNP R687C in exon 14 of *MYBL2* was identified in a genome scan study in Ashkenazi patients with type 2 diabetes (50), suggesting a potential role of MYBL2 in diabetes. In the present study, we found *MYBL2* showed an upregulated trend in T1DM patients compared with healthy controls (Supplementary Figure 4). In addition, we discovered that 40% of *hsa_circ_0060450* is present in the cytoplasm, with the remaining proportion in the nucleus, and in the current study we mainly focused on the role of cytoplasmic *hsa_circ_0060450*; however, it remains to be elucidated whether *hsa_circ_0060450* in the nucleus also functions in T1DM through the interaction with MYBL2.

In summary, in this study, we obtained a differential circRNA expression profile, and characterized a differentially expressed circRNA, *hsa_circ_0060450*. Subsequent experiments showed that *hsa_circ_0060450* can adsorb miR-199a-5p, releasing its target SHP2, by which *hsa_circ_0060450* inhibits macrophage-mediated inflammation through the suppression of JAK-STAT1/3 signaling pathway triggered by type I interferon. Overall, this study indicates the important role of *hsa_circ_0060450* in T1DM and implies a promising therapeutic molecule in the treatment of T1DM in the future.

## Data Availability Statement

All datasets generated for this study are included in the article/Supplementary Material.

## Ethics Statement

The studies involving human participants were reviewed and approved by Research Ethics Board of the Children's Hospital of Fudan University. Written informed consent to participate in this study was provided by the participants' legal guardian/next of kin.

## Author Contributions

LY, XH, CZ, CS, SH, WX, YG, QL, FL, and WL designed, carried out experiments, and analyzed data. LY and YZ wrote the manuscript. YZ and JF planned, designed, supervised, and coordinated the overall research efforts. All authors contributed to the article and approved the submitted version.

## Conflict of Interest

The authors declare that the research was conducted in the absence of any commercial or financial relationships that could be construed as a potential conflict of interest.
