# Supplementary material for: Hsa_circ_0060450 Negatively Regulates Type I Interferon-Induced Inflammation by Serving as miR-199a-5p Sponge in Type 1 Diabetes Mellitus
Source: Front Immunol. 2020 Sep 29;11:576903. doi: 10.3389/fimmu.2020.576903 (PMC7550460; doi:10.3389/fimmu.2020.576903)
Supplement: Supplementary file 1 [file Data_Sheet_1.pdf]

Supplementary materials

Hsa_circ_0060450 negatively regulates type I interferon-induced inflammation by serving as miR-199a-5p sponge in type 1 diabetes mellitus

Lan Yang[1,2#], Xiao Han[1,2#], Caiyan Zhang[1,2#], Chengjun Sun[3#], Saihua Huang[1,2], Wenfeng Xiao[1,2], Yajing Gao[1,2], Qiuyan Liang[1,2], Feihong Luo[3], Wei Lu[3], Jinrong Fu[1]* and Yufeng Zhou[1,2]*

[1] Institute of Pediatrics, Children's Hospital of Fudan University, and the Shanghai Key Laboratory of Medical Epigenetics, International Co-laboratory of Medical Epigenetics and Metabolism, Ministry of Science and Technology, Institutes of Biomedical Sciences, Fudan University, Shanghai 200032, China

[2] National Health Commission (NHC) Key Laboratory of Neonatal Diseases, Fudan University, Shanghai, China

[3] Department of Pediatric Endocrinology and Inherited Metabolic Diseases, Children's Hospital of Fudan University, Shanghai, 201102, China

Running title: *Hsa_circ_0060450* regulates inflammation in T1DM

# L.Y., X.H., C.Z., C.S. contributed equally to this work.

*To whom correspondence should be addressed:

Yufeng Zhou, M.D., Ph.D., 399 Wanyuan Rd, Minhang, Shanghai 201102, China.

E-mail: yfzhou1@fudan.edu.cn ; or Jinrong Fu， M.D., Ph.D., 399 Wanyuan Rd, Minhang, Shanghai 201102, China. E-mail：fujinrong@hotmail.com

Figure S1 The full length of *hsa_circ_0060450*.

hsa_circ_0060450 circRNA Mature Sequence
CGAGGATCTGGATGAGCTGCACTACCAGGACACAGATTCAGATGTGCCGGAGCAGAGGGAT
AGCAAGTGCAAGGTCAAATGGACCCATGAGGAGGACGAGCAGCTGAGGGCCCTGGTGAG
GCAGTTTGGACAGCAGGACTGGAAGTTCCTGGCCAGCCACTTCCCTAACCGCACTGACCAG
CAATGCCAGTACAGGTGGCTGAGAGTTTTGAATCCAGACCTTGTCAAGGGGCCATGGACCA
AAGAGGAAGACCAAAAAGTCATCGAGCTGGTTAAGAAGTATGGCACAAAGCAGTGGACAC
TGATTGCCAAGCACCTGAAGGGCCGGCTGGGGAAGCAGTGCCGTGAACGCTGGCACAACC
ACCTCAACCCTGAGGTGAAGAAGTCTTGCTGGACCGAGGAGGAGGACCGCATCATCTGCGA
GGCCCACAAGGTGCTGGGCAACCGCTGGGCCGAGATCGCCAAGATGTTGCCAGGGAG

Figure S2

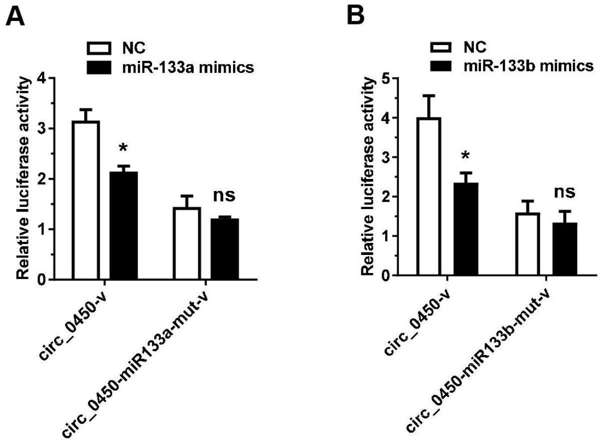

Figure S2 The target sequence of miR-133a or miR-133b in *hsa_circ_0060450* was confirmed by binding sites-mutant luciferase assay. (A) Luciferase reporter assay to detect the luciferase activity of 293T cells co-transfected with psicheck2 recombinant vector containing mutant *hsa_circ_0060450* fragment and miR-133a or NC. **(B)** Luciferase reporter assay to detect the luciferase activity of 293T cells co-transfected with psicheck2 recombinant vector containing mutant *hsa_circ_0060450* fragment and miR-133b or NC. NC, negative control. circ_0450-v, psicheck2 recombinant vector containing WT *hsa_circ_0060450* fragment. circ_0450-miR133a-mut-v, psicheck2 recombinant vector containing miR133a binding site-mutant *hsa_circ_0060450* fragment. circ_0450-miR133b-mut-v, psicheck2 recombinant vector containing miR133b binding site-mutant *hsa_circ_0060450* fragment. Data represent means ± SEM. *$p<0.05$. ns, not significance.

Figure S3

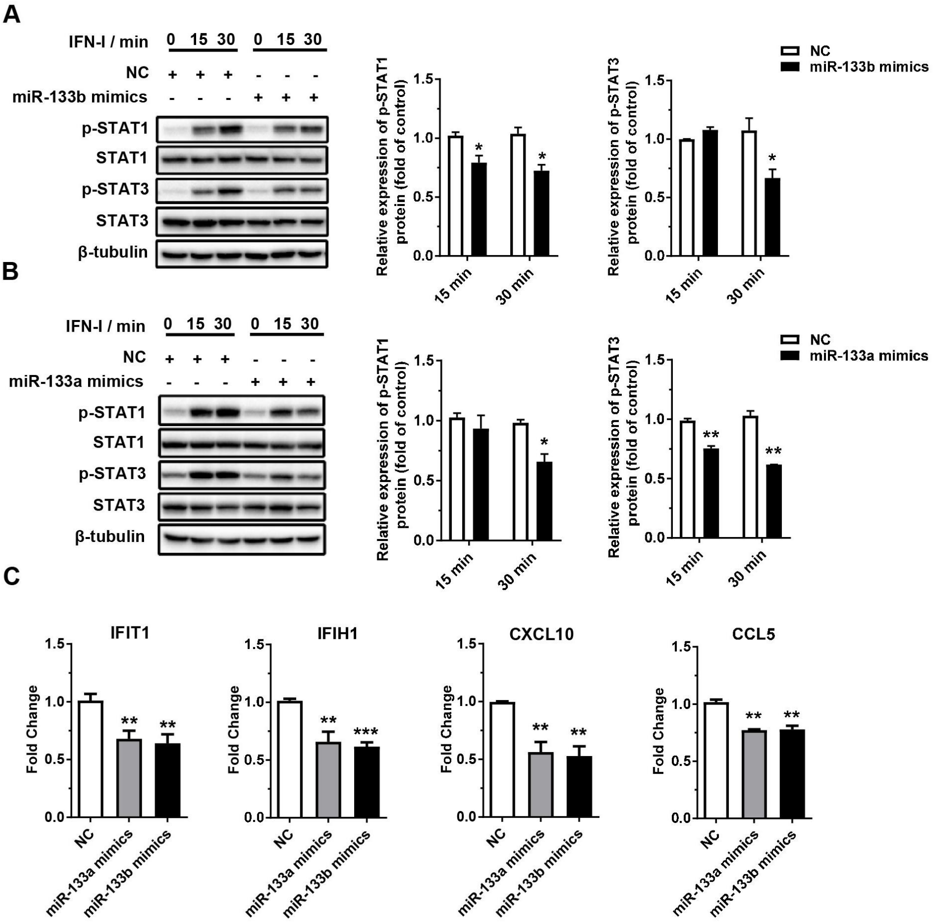

Figure S3 miR-133a and miR-133b inhibited the activation of IFN-I signal. (A) STAT1 and STAT3 protein phosphorylation assessment (left) and quantification analysis (right) at 15 min and 30 min of IFN-I stimulation after treatment with miR-133b mimics. **(B)** STAT1 and STAT3 protein phosphorylation assessment (left) and quantification analysis (right) at 15 min and 30 min of IFN-I stimulation after treatment with miR-133a mimics. **(C)** RT-qPCR analyses of *IFIT1*, *IFIH1, CXCL10*, and *CCL5* under IFN-I stimulation after treatment with miR-133a and miR-133b mimics. NC, negative control. Data represent means ± SEM. *p<0.05, **p<0.01, ***p<0.001.

Figure S4

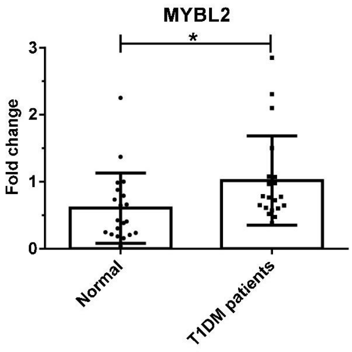

Figure S4 The expression of *MYBL2* is upregulated in T1DM patients. RT-qPCR analysis of the expression of *MYBL2* in the PBMCs of T1DM patients and healthy controls. Data represent means ± SEM. *p<0.05.

Table S1 Sequences of siRNAs and microRNA mimics

Name	Target RNA	Sense sequence (5′- 3′)
si-circ_0450-1	*hsa_circ_0060450*	GUUGCCAGGGAGCGAGGAUTT
si-circ_0450-2	*hsa_circ_0060450*	CAGGGAGCGAGGAUCUGGATT
si-SHP2	SHP2	GUUGACAAGAGGAGUUGAUTT
miR-199a -5p mimics	-	CCCAGUGUUCAGACUACCUGUUC
miR-133a-3p mimics	-	UUUGGUCCCCUUCAACCAGCUG
miR-133b mimics	-	UUUGGUCCCCUUCAACCAGCUA
NC	-	UUCUCCGAACGUGUCACGUTT

Table S2 Primer sequences used for amplification

Name	Usage	Sequence (5′- 3′)
β-actin	qPCR forward	CACCATTGGCAATGAGCGGTTC
	qPCR reverse	AGGTCTTTGCGGATGTCCACGT
circ_0450	qPCR forward	AGGGAGCGAGGATCTGGA
	qPCR reverse	CGGTTAGGGAAGTGGCTGG
MYBL2	qPCR forward	CACCAGAAACGAGCCTGCCTTA
	qPCR reverse	CTCAGGTCACACCAAGCATCAG
IFIT1	qPCR forward	GCCTTGCTGAAGTGTGGAGGAA
	qPCR reverse	ATCCAGGCGATAGGCAGAGATC
IFIH1	qPCR forward	GCTGAAGTAGGAGTCAAAGCCC
	qPCR reverse	CCACTGTGGTAGCGATAAGCAG
iNOS	qPCR forward	GCTCTACACCTCCAATGTGACC
	qPCR reverse	CTGCCGAGATTTGAGCCTCATG
CXCL10	qPCR forward	GGTGAGAAGAGATGTCTGAATCC
	qPCR reverse	GTCCATCCTTGGAAGCACTGCA
SHP2	qPCR forward	GACTTTTGGCGGATGGTGTTCC
	qPCR reverse	CGGCGCTTTCTTTGACGTTCCT
SHP1	qPCR forward	TTGACCACAGCCGAGTGATCCT
	qPCR reverse	CTGGCGATGTAGGTCTTAGCGT
SOCS1	qPCR forward	TTCGCCCTTAGCGTGAAGATGG
	qPCR reverse	TAGTGCTCCAGCAGCTCGAAGA
SOCS3	qPCR forward	CATCTCTGTCGGAAGACCGTCA
	qPCR reverse	GCATCGTACTGGTCCAGGAACT
PTP1B	qPCR forward	TGTCTGGCTGATACCTGCCTCT
	qPCR reverse	ATCAGCCCCATCCGAAACTTCC
circ_0450-v	PCR forward	CCGCTCGAGGTGAGGCAGTTTGGACAGC
	PCR reverse	ATTTGCGGCCGCGGCAACATCTTGGCGATC
circ_0450-miR	PCR forward	GAAGCAGTGCCGACTTGCGACCCACAACCACCTCA

199a-mut-v	PCR reverse	TGAGGTGGTTGTGGGTCGCAAGTCGGCACTGCTTC
circ_0450-miR	PCR forward	GACCAAAGAGGAACTGGTTTAAGTCATCGAGCTGG
133a/b-mut-v	PCR reverse	CCAGCTCGATGACTTAAACCAGTTCCTCTTTGGTC
SHP2-v	PCR forward	CCGCTCGAGTCAAGAACAGACGCAAGAA
	PCR reverse	ATTTGCGGCCGCAGCCAAACTACCCCAAAG
